# Multifunctional Bioactivity Electrospinning Nanofibers Encapsulating Emodin Provide a Potential Postoperative Management Strategy for Skin Cancer

**DOI:** 10.3390/pharmaceutics16091131

**Published:** 2024-08-27

**Authors:** Peiwen Ye, Reyisha Yusufu, Zhenfeng Guan, Tiantian Chen, Siyi Li, Yanping Feng, Xiaoyan Zeng, Jingya Lu, Muxiang Luo, Fenghuan Wei

**Affiliations:** 1 School of Traditional Chinese Medicine, Southern Medical University, Guangzhou 510515, China; ypwen@smu.edu.cn (P.Y.); reyishayusufu@smu.edu.cn (R.Y.); guan0769@i.smu.edu.cn (Z.G.); chentiantian@i.smu.edu.cn (T.C.); yuanshandamowang@i.smu.edu.cn (S.L.); fengyanping@i.smu.edu.cn (Y.F.); zxx22020294@i.smu.edu.cn (X.Z.); jingya@i.smu.edu.cn (J.L.); luomuxiang@i.smu.edu.cn (M.L.); 2 Guangdong Provincial Key Laboratory of Chinese Medicine Pharmaceutics, Guangzhou 510515, China

**Keywords:** emodin, electrospinning nanofiber, anti-cancer, anti-bacteria, skin cancer, postoperative management

## Abstract

Skin cancer is threatening more and more people’s health; its postoperative recurrence and wound infection are still critical challenges. Therefore, specialty wound dressings with multifunctional bioactivity are urgently desired. Emodin is a natural anthraquinone compound that has anti-cancer and anti-bacterial properties. Herein, we fabricated coaxial electrospinning nanofibers loaded with emodin to exploit a multifunctional wound dressing for skin cancer postoperative management, which encapsulated emodin in a polyvinylpyrrolidone core layer, combined with chitosan-polycaprolactone as a shell layer. The nanofibers were characterized via morphology, physicochemical nature, drug load efficiency, pH-dependent drug release profiles, and biocompatibility. Meanwhile, the anti-cancer and anti-bacterial effects were evaluated in vitro. The emodin-loaded nanofibers exhibited smooth surfaces with a relatively uniform diameter distribution and a clear shell-core structure; remarkably, emodin was evenly dispersed in the nanofibers with significantly enhanced dissolution of emodin. Furthermore, they not only display good wettability, high emodin entrapment efficiency, and biphasic release profile but also present superior biocompatibility and anti-cancer properties by increasing the levels of MDA and ROS in A-375 and HSC-1 cells via apoptosis-related pathway, and long-term anti-bacterial effects in a dose-independent manner. The findings indicate that the emodin-loaded nanofiber wound dressing can provide a potential treatment strategy for skin cancer postoperative management.

## 1. Introduction

Skin cancer is threatening more and more people’s health. Currently, the ideal treatment strategy is to excise all the skin cancerous tissues [1,2]. However, due to the complexity of the tumor microenvironment [3], it is hard to completely remove tumor cells, which inevitably leads to cancer recurrence [4]. Moreover, skin cancer patients undergoing surgical resection with reduced immunity are susceptible to bacterial infection [2,5], which not only could lead to tumor metastasis and recurrence but also could induce delayed wound closure [6]. Therefore, fabricating drug-loaded wound dressing with multifunctional properties to kill cancer cells and bacteria is very necessary and urgent [7,8].

Emodin, as a natural anthraquinone compound, is not only widely present in plants but also has outstanding pharmacological activities, such as anti-cancer, anti-oxidant, anti-inflammatory, and anti-microbial effects [9,10,11,12]. With the increasing incidence of tumors and the severity of anti-microbial resistance, emodin has attracted enormous attention based on its potent anti-tumor and anti-bacterial activities [13,14,15,16]. However, the application of emodin is hindered by low oral bioavailability induced by poor water solubility and extensive glucuronidation in the liver and intestine [17,18]. To improve its bioavailability, multiple emodin-loaded delivery systems have been developed, such as thermo-reversible gel [19], nanoemulsion [20], microspheres [21], and electrospinning nanofibers [10,22], among which electrospinning nanofibers obviously are a better choice for wound dressing based on their unique characteristics, such as large specific surface and high porosity favorable for absorbing wound exudate and maintaining normal air communication with outside, good bionic properties similar to natural extracellular matrix [23,24]. Remarkably, expectant drug release profiles of nanofibers could be fabricated by optimizing electrospinning conditions [10]. Therefore, emodin-loaded nanofiber wound dressings are a promising candidate for the application of skin cancer treatment.

Presently, a monoaxial electrospinning wound dressing loaded with emodin achieved a rapid cumulative release of up to 96.7% in the first 60 min has been reported, which would lead to the nanofiber membranes being replaced frequently and disturbing wound healing [22]. In our previous study, the coaxial electrospinning nanofibers encapsulating emodin in the polyvinylpyrrolidone (PVP) core layer with cellulose acetate (CA) shell layer were fabricated, in which emodin from the nanofibers displayed a sudden release within 0.5 h reaching 49.15%, and then shifted to a steady release reaching 100% for the next 6 days, obviously indicating the advantages of coaxial electrospinning mode in controlling drug release [10]. Moreover, matrix materials play a critical role in the drug delivery system. Chitosan (CS) is a unique alkaline polysaccharide in nature, which exhibits protonation and solubilization to a greater extent in acidic tumor environments of pH between 6.0 and 6.5 [25] and can efficiently interact with the membranes of cancer cells and endothelial cells of tumor vasculature characterized by overexpressed anionic surface moieties [26]. Remarkably, chitosan presents minimal toxicity towards non-cancerous cells [27] and has been used in anti-cancer therapeutics and wound healing because of its biocompatibility, anti-cancer and anti-bacterial properties [28,29,30]. Nevertheless, our previous study and published references demonstrated that the electrospinnability of chitosan is very limited because of its cationic nature [31]. In addition, it is poorly soluble in aqueous and organic media because of its high crystallinity. Thus, it is necessary to blend with other polymers to enhance its electrospinnability. PVP is an important hydrophilic polymer that exerts excellent electrospinnability and properties in enhancing the water solubility of hydrophobic drugs and inhibiting the recrystallization of crystalline drugs [10,32]. However, PVP can not control emodin release because of its hydrophilic properties [22]. Polycaprolactone (PCL), as a semi-crystalline hydrophobic polymer possessing good mechanical properties and electrospinnability, is commonly used as a biomedical polymer for controlling drug release [33,34]. Therefore, based on the respective properties of CS, PCL, and PVP, fabricating emodin-loaded CS-PCL-PVP electrospinning wound dressing can not only kill skin cancer cells and pathogenic bacteria but also maximize their advantages and remedy their respective disadvantages [35].

Herein, a nanofiber drug delivery system encapsulating emodin in the PVP core layer, combined with CS-PCL as a shell layer, was fabricated by coaxial electrospinning under optimized conditions. Meanwhile, the diameter, morphology, physical and chemical properties, wettability, swelling ratio, and drug-loading content of the emodin-loaded nanofibers were examined. Furthermore, the biphasic release profile of emodin from the nanofibers at different pH conditions was evaluated, and the cytocompatibility was determined with three types of cell lines (HSF, HaCaT, and HUVEC cells); the anti-cancer effect and underlying mechanism were confirmed with two tumor cell lines (A-375 and HSC-1 cells), and the anti-bacterial activities were determined with three types of bacteria (Staphylococcus aureus, Staphylococcus hominis, and Staphylococcus epidermidis). Altogether, the findings demonstrated that wound dressing with emodin would be a promising candidate for postoperative skin cancer management.

## 2. Materials and Methods

### 2.1. Materials

Emodin (purity, 98.6%) was purchased from WeiKeQi Biological Technology Co., Ltd. (Chengdu, China). Polyvinylpyrrolidone (PVP, Mw ~1,300,000 g/mol), polycaprolactone (PCL, Mw ~80,000), and chitosan (CS, >400 mPa·s) were purchased from Dalian Meilun Biological Technology Co., Ltd. (Dalian, China). HaCaT, HSF, HUVEC, A-375, and HSC-1 cells were purchased from the China Center for Type Culture Collection (CCTCC, Wuhan, China). *Staphylococcus aureus* (*S. aureus*), *Staphylococcus hominis* (*S. hominis*), and *Staphylococcus epidermidis* (*S. epidermidis*) were collected from Southern Hospital, Southern Medical University, Guangzhou, China.

### 2.2. Preparation of Coaxial Electrospinning Nanofibers

First, the solutions of the core and shell layers were prepared, respectively. CS was dissolved in 50% acetic acid aqueous solution by magnetic stirring to obtain 1–3% CS solution (*w*/*v*). PCL was dissolved in a mixture solvent of formic acid and acetic acid (*v*/*v*) by magnetic stirring to obtain 10–20% PCL solution (*w*/*v*). Subsequently, the different ratios of CS-PCL (*v*/*v*) were prepared to obtain the corresponding shell layer solution. PVP was dissolved in absolute ethanol by magnetic stirring to obtain 10–18% PVP solution (*w*/*v*), and then emodin (2, 4, and 7.5% relative to PVP, *w*/*w*) was added into PVP solution by vortex to obtain a homogeneous core layer solution.

Herein, the electrospinning equipment (Ucalery Co., Beijing, China) is mainly composed of two syringe pumps with a coaxial metallic needle of 17 G (1.12 mm inner diameter and 1.48 mm outer diameter), a high-voltage power supply (0–30 kV), and a drum collector.

The electrospinning was carried out according to the procedures described previously [10]. First, the shell and core layer solutions were transferred into a 2.5 mL syringe, respectively. Following, 10.0–28.0 kV of voltage, 0.1–0.4 mm/min flow rates of the core solution and shell solution, 15–20 cm of distances between the nozzle and the collector were performed to optimize ideal electrospinning conditions, and a square aluminum foil (15 × 30 cm) was used as receiving carrier placed on collector rotating at 500 rpm. All electrospinning experiments were carried out at room temperature with 48–67% relative humidity, and then the nanofiber membranes were dried in a glass desiccator with silica-gel desiccant over 72 h for further application.

### 2.3. Characterization of Electrospinning Nanofibers

#### 2.3.1. Morphologies

To observe the morphology of the nanofibers under a scanning electron microscope (SEM), the nanofiber membranes were covered with 5 nm Au/Pd by a sputtering machine (Cressington Auto 108 Sputter Coater, Ted Pella Inc., Redding, CA, USA), and then morphologies and diameters of the nanofibers were observed by the SEM (EVO Sem, Ma10. Zeiss, Oberkochen, Germany). Meanwhile, the images were photographed at 500–5000 magnification. The diameters of the nanofibers were measured using Image J software (ver.1.8.0), and the diameter distribution histograms were generated by Origin software (ver.8.0).

To simulate the morphological change in the emodin-loaded nanofibers after absorbing tissue fluid, the nanofibers were immersed in PBS buffer solution (pH 7.4) for 4 h. Subsequently, the nanofibers were sucked away the fluid using filter paper and were covered with 5 nm Au/Pd, and then the morphologies of the nanofibers were observed by the SEM.

In addition, to identify the core-shell structure of the nanofibers, put them into a 200 × 200 Copper Mesh and observed their structure by applying a transmission electron microscope (TEM, HT7800, Hitachi, Nagano, Japan) at an accelerating voltage of 120 kV in a bright field mode.

#### 2.3.2. Attenuated Total Reflection–Fourier Transform Infrared Spectra Analysis

To analyze the characteristic functional groups of the nanofibers without destroying material structures, attenuated total reflection (ATR) was used according to the previous study [10]. The nanofibers with or without emodin and raw materials, including emodin, PVP, CS, and PCL, were analyzed using a Fourier transform infrared spectra (FTIR) instrument along with ATR (Bruker Tensor II, Karlsruhe, Germany) in the 400–4000 cm^−1^ range with a resolution of 4 cm^−1^.

#### 2.3.3. X-ray Diffraction

To check the existing form of emodin in the nanofibers and to better examine the property of the nanofibers loading emodin, the nanofiber membranes and the raw materials were analyzed with X-ray diffraction (XRD, Empyrean, PANalytical, Almelo, The Netherlands) under 3–60° 2θ range with 10°/min scan rate of the diffracted intensity under Cu Kα radiation.

#### 2.3.4. Differential Scanning Calorimetry

To further evaluate the status of emodin in the nanofibers, differential scanning calorimetry (DSC, DSC21400A-0211-L, Netzsch, Selb, Germany) was used to detect the nanofibers with or without emodin and the raw materials at 20 to 300 °C with a heating rate of 10 K/min under nitrogen purging.

#### 2.3.5. Wettability and Swelling Ratio

The wettability of a wound dressing is one of the most important parameters, which is usually assessed by water contact angle [36]. To measure the wettability of the emodin-loaded nanofiber membrane, a drop shape analyzer (DSA25, KRUSS, Hamburg, Germany) was used. A volume of 3 μL distilled water was dropped on the nanofiber membranes, and then the contact angles were recorded at 0, 5, 10, and 20 s, respectively.

The swelling ratio is another key parameter for evaluating the capacity of wound dressings to absorb wound exudate, which is usually determined using the gravimetric method [37,38]. Briefly, the nanofiber membranes were cut into 3 × 3 cm, weighed accurately in triplicate, and then were immersed into 20 mL of PBS (pH = 7.4, 25 ± 2 °C). At presupposed intervals (0.5, 1, 2, 4, 6, 8, 12, 24, and 48 h), the membranes were taken out, and then the water adhered to the membrane surface was removed before being weighed. The swelling ratio was calculated using the following formula: Swelling ratio (%) = (Swollen weight − Initial dried weight) × 100/Initial dried weight.

#### 2.3.6. Encapsulation Efficiency and Drug Release Profiles

The encapsulation efficiency of emodin in the nanofibers was determined according to the procedures described previously [10]. Briefly, the emodin-loaded nanofiber membranes were weighed accurately in triplicate and were extracted in methanol using an ultrasonic device (KQ-100, KunShan Ultrasonic Instruments Co., Ltd., Kunshan, China) for 30 min, and then the extract solution was centrifuged at 10,000 rpm for 10 min. Subsequently, the supernatant was analyzed using a UV/vis spectrophotometer (UV-Vis 8454, Agilent, Santa Clara, CA, USA) at 254 nm. To avoid background interference, the methanol solution of the nanofibers without emodin was selected as the control sample. The quantity of emodin released from nanofibers was assessed using a standard curve of emodin reference sample analyzed in the same conditions. Encapsulation efficiency was calculated with the following equation:

Encapsulation efficiency (%) = (Determined content in nanofibers/Added content in electrospinning solution) × 100.

The release profiles of emodin from the nanofibers at different pH solutions were determined following this procedure: The nanofiber membranes and emodin raw material were incubated in 50 mL PBS solution (pH 5.0, 7.4 and 8.5) at 37 ± 1 °C, respectively. The aliquots were collected from the release medium at designated intervals of 0.083, 0.17, 0.5, 1, 2, 4, 6, 8, 12, 24, 30, 42, and 48 h and presupposed days; meanwhile, fresh PBS of the same volume was replaced. Subsequently, the sample solutions were centrifuged at 10,000 rpm for 10 min at room temperature, and the supernatant was determined by UV/vis spectrophotometer at 254 nm, respectively. The emodin released from the nanofibers was calculated using the standard curve of emodin determined in the same conditions. All measurements were performed in triplicate.

### 2.4. Cytocompatibility

To evaluate the cytocompatibility of the emodin-loaded nanofibers, the viabilities of human skin fibroblast (HSF) cells, human keratinocyte HaCaT (HaCaT) cells, and human umbilical vein endothelial cells (HUVEC) treated with the nanofibers were examined by CCK-8 assays (Dojindao, Kyushu Island, Japan). HSF, HaCaT, and HUVEC cells were cultured according to the previous method [10]. A quantity of 5 × 10^3^ cells was seeded into each well in a 96-well plate (Corning, New York, NY, USA) and then cultured overnight. The nanofiber membranes (loading 0.0824, 0.169, and 0.317 μg emodin, sanitized by UV irradiation for 30 min before use) were put into the 96-well plates with the cells and then inoculated together. Each group performed three replicated wells. Cell morphologies were viewed using an inverted microscope (Nikon, Tokyo, Japan). Subsequently, 10 µL of CCK-8 reagent (Zeta Life, San Mateo, CA, USA) was put into each well and then incubated at 37 °C for 1 h on days 0, 1, 2, 3, 4, 5, 6, and 7, respectively. On each day, OD_450_ values were measured using a microplate reader (Multiskan GO, Thermo FC, Waltham, MA, USA).

Collective cell migration is a hallmark of wound repair [39]. Herein, a wound healing assay was used to evaluate the effects of the emodin-loaded nanofibers on the migration abilities of HSF, HaCaT, and HUVEC cells. A quantity of 1.0 × 10^5^ cells per well was seeded in a 12-well plate and incubated for 24 h at 37 °C in a humidified atmosphere containing 5% CO_2_. Following, scratch wounds were created using a sterile 200 μL pipette tip, and then the nanofiber membranes (loading 1.648, 3.38, and 6.34 μg emodin, sanitized using UV irradiation for 30 min before use) were put into the 12-well plate with cells and inoculated together for 24 h. The inverted microscope was used to obtain images at 0 and 24 h, respectively. The changes in the scratched area were measured using Image J software (ver.1.8.0), and the migration rates of the cells were calculated by the following equation:

Scratch healing rate (%) = (1 − A_i_/A_0_) × 100%,

where A_0_ and A_i_ are the scratch areas before and after different treatments, respectively.

### 2.5. Anti-Cancer Effects

To evaluate the anti-tumor activities of the emodin-loaded nanofibers, the viabilities of A-375 human melanoma cells (A-375 cells) and HSC-1 human cutaneous squamous cell carcinoma (HSC-1 cells) treated with the nanofibers were detected by CCK-8 assays. A-375 and HSC-1 cells were cultured according to the previous method [10]. A quantity of 5 × 10^3^ cells was seeded into each well in a 96-well plate and cultured overnight. The nanofiber membranes (loading 0.0824, 0.169, and 0.317 μg emodin, sanitized by UV irradiation for 30 min before use) were put into the 96-well plates with the cells and then inoculated together. Each group performed three replicated wells. Cell morphologies were viewed using the inverted microscope. Subsequently, 10 µL of CCK-8 reagent was put into each well and then incubated at 37 °C for 1 h on days 0, 1, 2, 3, 4, 5, 6, and 7, respectively. On each day, the OD_450_ values were measured using the microplate reader.

A wound-healing assay was used to evaluate the inhibitory effects of the nanofibers on the metastasis of A-375 and HSC-1 cells. A quantity of 2 × 10^5^ cells per well was seeded in a 12-well plate and incubated for 24 h at 37 °C in a humidified atmosphere containing 5% CO_2_. First, scratch wounds were created using a 200 μL pipette tip, and then the nanofiber membranes (loading 1.648, 3.38, and 6.34 μg emodin, sterilized using UV for 30 min before use, respectively) were transferred into the 12-well plates and inoculated together for 24 and 48 h, respectively. The images at 0 and 24 h were obtained using the inverted microscope, respectively. The changes in the scratched area were measured using Image J software (ver.1.8.0), and the migration rates of the cells were calculated by the following equation: Scratch healing rate (%) = (1 − A_i_/A_0_) × 100%, where A_0_ and A_i_ are the scratch areas before and after different treatments, respectively.

To further detect the anti-cancer effects of the nanofibers, the invasion activity of A-375 and HSC-1 treated with the nanofibers was assessed using a transwell assay. A quantity of 1 × 10^5^ cells per well was added to the upper chamber of the 24-well transwell plate and starved for 24 h to form a cell monolayer. The different culture mediums with or without the nanofiber membranes (loading 0.412, 0.845, and 1.585 μg emodin, sterilized using UV for 30 min before use, respectively) were immersed in the upper chambers and co-incubation for 48 h at 37 °C. Subsequently, the cells that invaded the lower surface areas were fixed with 4% paraformaldehyde for 20 min and then stained with 0.1% crystal violet for 30 min. Afterward, five fields were selected randomly and counted under a microscope.

### 2.6. Mechanism of Anti-Cancer Effects

#### 2.6.1. Cell Death Inhibitors

To predict the potential mechanism of the emodin-loaded nanofibers in inhibiting the proliferation of A-375 and HSC-1 cells, cell death inhibitors including Ferrostatin-1 (a ferroptosis inhibitor, MedChemExpress, Monmouth Junction, NJ, USA), Z-VAD(OMe)-FMK (an apoptosis inhibitor, MedChemExpress, Monmouth Junction, NJ, USA), Necrosulfonamide (a necroptosis inhibitor, MedChemExpress, Monmouth Junction, NJ, USA), and MRT68921 (an autophagy inhibitor, MedChemExpress, Monmouth Junction, NJ, USA) were used. A-375 and HSC-1 cells were cultured according to the previous method [10]. A quantity of 1 × 10^3^ cells per well was seeded in a 96-well plate and cultured overnight. Subsequently, the nanofiber membranes (loading 0.634 μg emodin, sanitized by UV irradiation for 30 min before use) were put into the 96-well plates with the cells. Meanwhile, 5 μM Ferrostatin-1, 5 μM Z-VAD(OMe)-FMK, 1 μM Necrosulfonamide and 1 μM MRT68921 were added into the 96-well plates with the cells and nanofibers, respectively, and then inoculated together for 48 h. Following, 10 µL of CCK-8 reagent was added into each well and incubated at 37 °C for 1 h. Subsequently, OD_450_ values were measured using the microplate reader. Each group received three replicated wells.

#### 2.6.2. MDA Level

The malondialdehyde (MDA) level was measured using the lipid peroxidation MDA assay kit. A quantity of 1 × 10^6^ cells per well was seeded in a 6-well plate and incubated for 24 h at 37 °C in a humidified atmosphere containing 5% CO_2_. Subsequently, the cells were incubated with or without Z-VAD(OMe)-FMK and Necrosulfonamide, and the nanofiber membranes (loading 7.925 μg emodin, sanitized using UV irradiation for 30 min before use) for 48 h, respectively. Subsequently, 200 μL cell lysis buffer was added to the cells and incubated at 4 °C for 10 min, and then lysed for 30 min on ice. Supernatant lysis buffer was collected after centrifugation for 10 min at 12,000× *g* and 4 °C. Subsequently, 100 μL of lysis solution was mixed with 200 μL of malondialdehyde solution (S0131, Beyotime, Shanghai, China), and then incubated for 15 min at 100 °C water bath protected from light. 200 μL supernatant was transferred to a 96-well plate and then immediately measured absorbance at OD_532_ nm. Meanwhile, the standard curve was carried out according to the manufacturer’s instructions.

#### 2.6.3. ROS Level

Reactive oxygen species (ROS) were detected by 2′,7′-dichlorodihydrofluorescein diacetate (DCFH-DA) staining in vitro. A quantity of 1 × 10^6^ cells per well was seeded in a 6-well plate and incubated for 24 h at 37 °C in a humidified atmosphere containing 5% CO_2_. Subsequently, the cells were incubated with or without Z-VAD(OMe)-FMK and Necrosulfonamide, and the nanofiber membranes were added (loading 7.925 μg emodin, sterilized using UV for 30 min before use, respectively) for 48 h, respectively. Following, the cells were stained with 10 μM DCFH-DA (Elabscience, Wuhan, China) in the dark for 1 h at 37 °C. The cells were then washed three times with PBS before the fluorescence was measured using a fluorescence microscope (excitation wavelength: 488 nm; emission wavelength: 525 nm).

### 2.7. Anti-Bacterial Activities

To investigate the anti-bacterial properties of the emodin-loaded nanofibers, methicillin-resistant *Staphylococcus aureus* (*S. aureus*), *Staphylococcus epidermidis* (*S. epidermidis*) and *Staphylococcus hominis* (*S. hominis*) were selected. The nanofiber membranes were cut into 6 mm diameter discs using a puncher and were disinfected under ultraviolet irradiation for 30 min before use, respectively. The bacteria (1.0 × 10^8^ CFU/mL) were incubated in nutrient broth on a shaker at 37 °C for 6 h and inoculated evenly on a Nutrient Agar plate, and then the nanofiber membranes containing 0, 30, and 210 μg emodin were placed on the plates, and incubated at 37 °C for 24, 48, and 72 h, respectively.

### 2.8. Statistical Analysis

All experimental data were expressed as mean ± standard deviation (SD). The statistical analysis was conducted using SPSS 20.0 software (International Business Machines Corp., Armonk, NY, USA). One-way analysis of variance (ANOVA) followed by the Bonferroni test was applied to evaluate the statistical significance, and a value of *p* < 0.05 was set as statistically significant.

## 3. Results and Discussion

### 3.1. Optimized Electrospinning Conditions

Electrospinning parameters play a critical role in the properties of resultant nanofiber. Thus, the conditions for fabricating the emodin-loaded nanofibers were carefully optimized according to the procedures described previously [10]. First, considering the high hydrophobicity of emodin, electrospinnability of the core and shell layer solutions, and morphological characteristics of the nanofibers, different matrix ratios among PVP, PCL and CS, and different solvents including anhydrous ethanol, acetone, DMAc, mixture of formic acid and acetic acid (7:3, 1:1, 3:7, *v*/*v*) to prepare the core and shell solutions, were successively tried. The optimal conditions were as follows: the mixture with 2:1 ratio (*v*/*v*) of 20% PCL dissolved in mixture solution of formic acid and acetic acid (7:3, *v*/*v*)—2% CS dissolved in 50% acetic acid aqueous solution as shell-layer matrix, 15% PVP containing up to 7.5% emodin dissolved in absolute ethanol as core layer solution; the flow rates of the core solution and the shell solution were 0.4 mm/min and 0.2 mm/min, respectively; the voltage was 14.5–15.5 kV; the receiving distance and rotation rate of the collector were 15 cm and 500 rpm, respectively. All electrospinning experiments were carried out at 45–50 °C with a relative humidity of 12–18%. The nanofiber membranes were first dried in a 40 °C baking oven for 12 h to remove the acetic and formic acid residues and then put in a glass desiccator with a silica-gel desiccant at room temperature before use. All the nanofibers discussed in this study were prepared using the optimum electrospinning conditions.

### 3.2. Characterization

#### 3.2.1. Morphology

**Figure 1:**
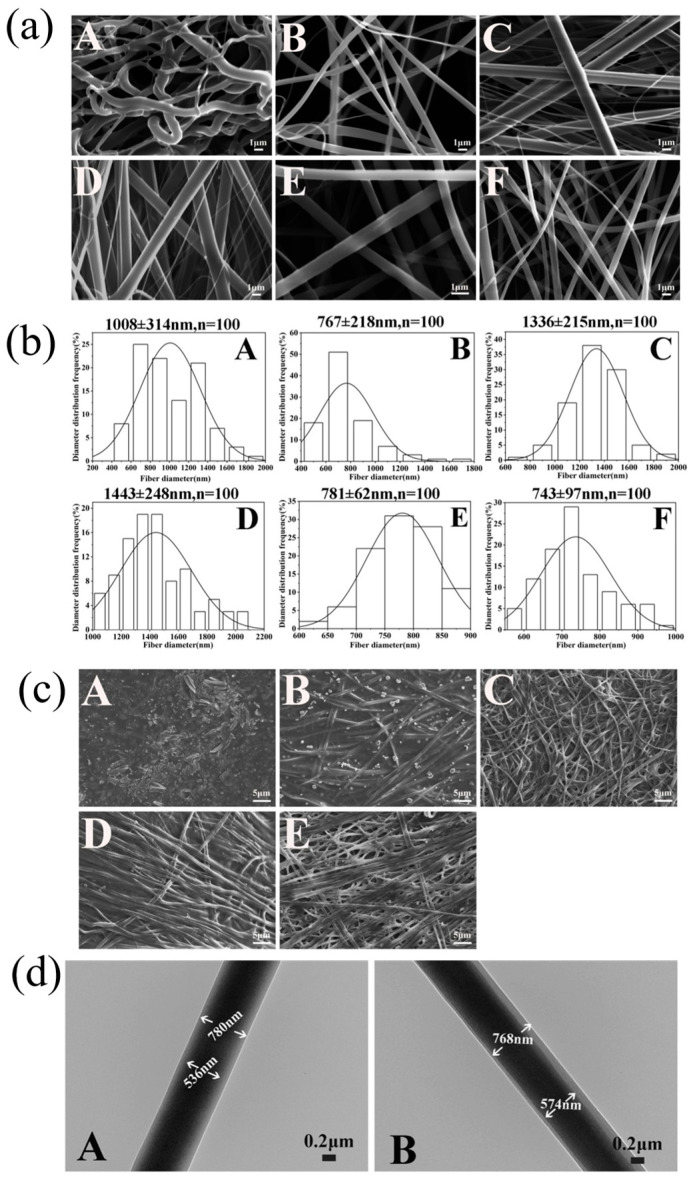
Morphology and diameter distributions of the nanofibers. (**a**) SEM images and (**b**) diameter distributions of nanofibers of 1% CS-emodin/13% PVP (**A**), 8% PCL/2% CS (3:2)-emodin/13% PVP (**B**), 16% PCL/2% CS (5:4)-emodin/13% PVP (**C**), 20% PCL/2% CS (3:1)-emodin/15% PVP (**D**), 20% PCL/2% CS (2:1)-emodin/15% PVP (**E**), 20% PCL/2% CS (2:1)–15% PVP without emodin (**F**). (**c**) SEM images of nanofibers after exposure to PBS solution (pH 7.4) of 8% PCL/2% CS (3:2)-emodin/13% PVP (**A**), 16% PCL/2% CS (5:4)-emodin/13% PVP (**B**), 20% PCL/2% CS (3:1)-emodin/15% PVP (**C**), 20% PCL/2% CS (2:1)-emodin/15% PVP (**D**), 20% PCL/2% CS (2:1)–15% PVP without emodin (**E**). (**d**) TEM images of 20% PCL/2% CS (2:1)-emodin/15% PVP (**A**), 20% PCL/2% CS (2:1)–15% PVP without emodin (**B**).

Morphology and diameter distributions of the nanofibers were displayed in Figure 1, which indicated that chitosan alone as the shell layer solution produced bent and uneven fibers with an average diameter of 1008 ± 314 nm and a wide distribution range (shown in Figure 1a(A),b(A)). With the addition of PCL solution into the CS shell layer solution, the fibers became smoother and straighter. With an increase in PCL concentration, the electrospinnability of the shell layer solution was enhanced; thus, the fiber morphology became uniform and smooth, and the fiber diameters decreased (shown in Figure 1a(B–F),b(B–F)). When PCL concentration was increased to 20% and mixed with 2% CS with a 2:1 ratio (*v*/*v*) as the shell layer, the fibers were the most homogeneous, and the average diameters of the nanofibers with emodin and without emodin were 781 ± 62 nm and 743 ± 97 nm, respectively. In addition, the nanofiber membranes exhibited good porous structure due to the small diameter of the fibers and the gaps among them, facilitating gas exchange between the wound area and outside, which is beneficial to wound healing [23].

The morphology of the nanofibers after absorbing PBS buffer solution (pH 7.4) for 4 h is shown in Figure 1b(A–E), which illustrates that the nanofibers swelled slightly with a certain degree of synechia. Remarkably, as the PCL concentration increased, the nanofibrous morphology still presented obviously, such as the nanofibers with 20% PCL–2% CS (2:1) as shell layer (shown in Figure 1b(D/E)), illustrating that the increased PCL concentration is a key parameter in maintaining the fiber structure of the emodin-loaded nanofibers in PBS buffer solution, which is essential for the proliferation and migration of normal cells [23]. This also demonstrates that the optimized emodin-loaded nanofibers possess great potential in improving wound healing.

The core-shell structure of the nanofibers is shown in Figure 1c(A–B), which illustrates that the nanofibers display obviously core-shell structure, and the core layer diameters of the nanofibers with emodin and without emodin were 446.68 ± 106.81 nm and 417.82 ± 30.54 nm, respectively, that were about two third of the whole diameter due to the 2:1 flow ratio of the core layer solution and shell layer solution.

#### 3.2.2. ATR-FTIR Spectra Analysis

**Figure 2:**
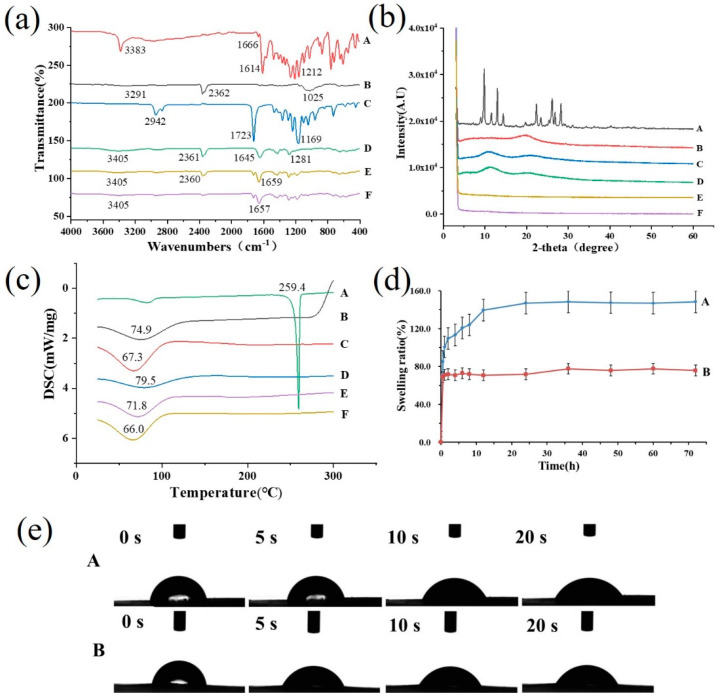
Characterization of nanofibers. (**a**) ATR–FTIR spectra, (**b**) XRD patterns, and (**c**) DSC themograms of emodin (A), CS (B), PCL (C), PVP (D), nanofibers with emodin (E), nanofibers witout emodin (F). (**d**) Swelling ratio of nanofibers with emodin (A) and without emodin (B) after exposure to PBS solution (pH 7.4). (**e**) Contact angles of nanofibers with emodin (**A**) and nanofibers without emodin (**B**) at 0, 5, 10, and 20 s, respectively.

The FTIR spectrum of the emodin raw material had two characteristic sharp peaks at 1614 cm^−1^ and 3383 cm^−1^ (shown in Figure 2a(A)), which is consistent with the standard infrared spectra of this compound and previous study [10], indicating the existence of emodin crystalline. A sharp peak at 1723 cm^−1^ in the FTIR spectrum of PCL was present because of its semi-crystalline character (shown in Figure 2a(C)). However, the characteristic peaks of emodin and PCL materials disappeared in the nanofibers with emodin, and most of the peaks in the fingerprint area were different from those of emodin and PCL (shown in Figure 2a(E)). Obviously, the results demonstrated that emodin was distributed in the nanofibers in a molecular state instead of a crystalline state because of the hydrogen bonding between PVP and emodin, also implying the stability of emodin in the nanofiber drug delivery system.

#### 3.2.3. X-ray Diffraction

The XRD pattern of the emodin raw material exhibited several intense and sharp diffraction peaks (shown in Figure 2b(A)), illustrating the crystalline form of emodin [10]. Remarkably, no obvious diffraction peaks appeared in the emodin-loaded nanofibers (shown in Figure 2b(E)), suggesting that the strong intermolecular interactions between emodin and the matrix-induced transformation of emodin from a crystalline to an amorphous state, which is consistent with the results of FTIR spectra analysis, and also confirmed the high dispersibility of emodin in the electrospinning nanofibers.

#### 3.2.4. Differential Scanning Calorimetry

The DSC thermogram of the emodin raw material exerted a sharp endothermic peak at 259.4 °C, while this characteristic peak of emodin disappeared in the emodin-loaded nanofibers (shown in Figure 2c(A/E)), which was identical to the previous result [10], also demonstrating that emodin crystalline was converted into an amorphous state, which further clearly verified the observation and conclusion from XRD and FTIR data.

#### 3.2.5. Swelling Ratio and Contact Angle

As shown in Figure 2d, the emodin-loaded nanofibers exhibited relatively high swelling ratios during 24 h, with a 147% swelling ratio compared with 77.7% of the nanofibers without emodin, that could be attributed to increased hydrogen bonding between emodin and water molecules [40].

The images of contact angles taken at 0, 5, 10, and 20 s after water dripped onto the surface of the nanofibers were shown in Figure 2e, which illustrated that the contact angles declined gradually from 75.28 ± 17.55° to 50.48 ± 2.38° with prolonging exposure time from 0 s to 20 s, suggesting the nanofiber membranes have good wettability and preferable adhesion for cells [7,41]. The swelling property and hydrophilicity demonstrated that the emodin-loaded nanofibers as wound dressing could absorb excess exudates and maintain a beneficial wound environment to facilitate wound repair.

#### 3.2.6. Encapsulation Efficiency and Release Profiles

**Figure 3:**
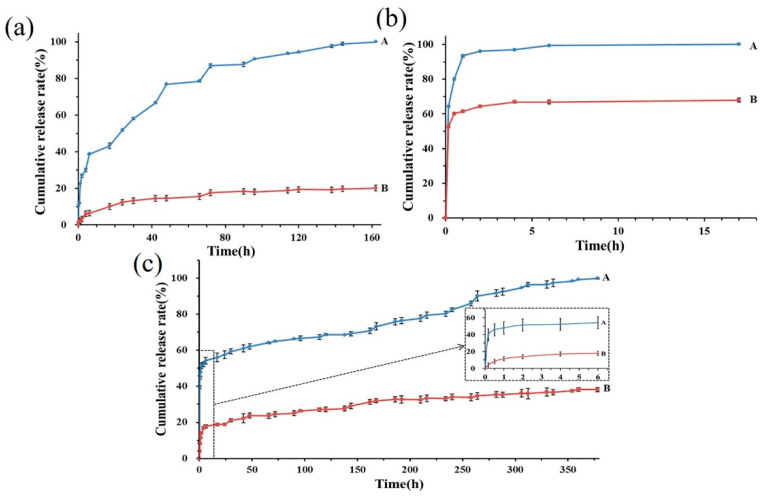
Cumulative release profiles of emodin in different pH PBS solutions. Release profiles of emodin from the nanofibers (A) and emodin raw materials (B) in PBS solution at pH 5.0 (**a**), pH 8.5 (**b**), and pH 7.4 (**c**), respectively.

The average entrapment efficiency of emodin in the nanofibers was 98.48 ± 0.31%, indicating that this drug delivery system fabricated by coaxial electrospinning technology had superior entrapment efficiency, which is identical to a previous study [10]. As shown in Figure 3, not only the cumulative release profiles of emodin from the nanofibers in PBS solution at pH 5.0, 7.4, and 8.5 were obviously different, but also the release rates between the nanofibers and emodin raw material in the same PBS solution were significantly distinct. Remarkably, in PBS solution at pH 8.5, the release rates of emodin from the nanofibers were the fastest, such as within 4 and 17 h, 97.0 ± 3.75% and 100% ± 3.87% of emodin was released, respectively, while only 60.2 ± 5.79% and 67.0 ± 5.19% of the emodin raw material was dissolved in the same period, which obviously demonstrated that the electrospinning nanofibers significantly promoted water dissolution of emodin. In PBS solution at pH 7.4, the cumulative release rate of the emodin raw material was 38.1 ± 2.02% within 378 h, while the nanofibers loading emodin reached 51.30 ± 7.92% rapid release within 2 h. Subsequently, a slow release occurred until complete release at 378 h, illustrating that emodin from the nanofibers exhibited a typical biphasic release profile at pH 7.4 PBS solution, which could be attributed to the hydrophilicity of the PVP matrix in the core layer and the hydrophobicity of the CS and PCL matrix in shell layer. In PBS solution at pH 5.0, the release rates of emodin were relatively slow, 43.19 ± 1.92% of emodin from the nanofibers was released within 17 h, subsequently, exhibited a slow release until complete release at 162 h, while only 20.1 ± 1.32% of the emodin raw material was dissolved in the same period. Altogether, the results suggest that the electrospinning nanofibers also significantly enhanced the solubility of emodin in the acidic medium compared with the emodin raw material, which is more beneficial to emodin for presenting anti-tumor activities in acidic tumor microenvironment [25], which also confirmed that the emodin-loaded nanofibers could be a promising candidate for the application of skin cancer treatment. Furthermore, the biphasic drug release profile of wound dressing is essential in large-area wound repair, which can reduce pain induced by frequent dressing changes, kill pathogenic bacteria rapidly upon treatment with the wound dressing, and prolong therapeutic effects through sustained release over a long term.

### 3.3. Cytocompatibility with Normal Cells

**Figure 4:**
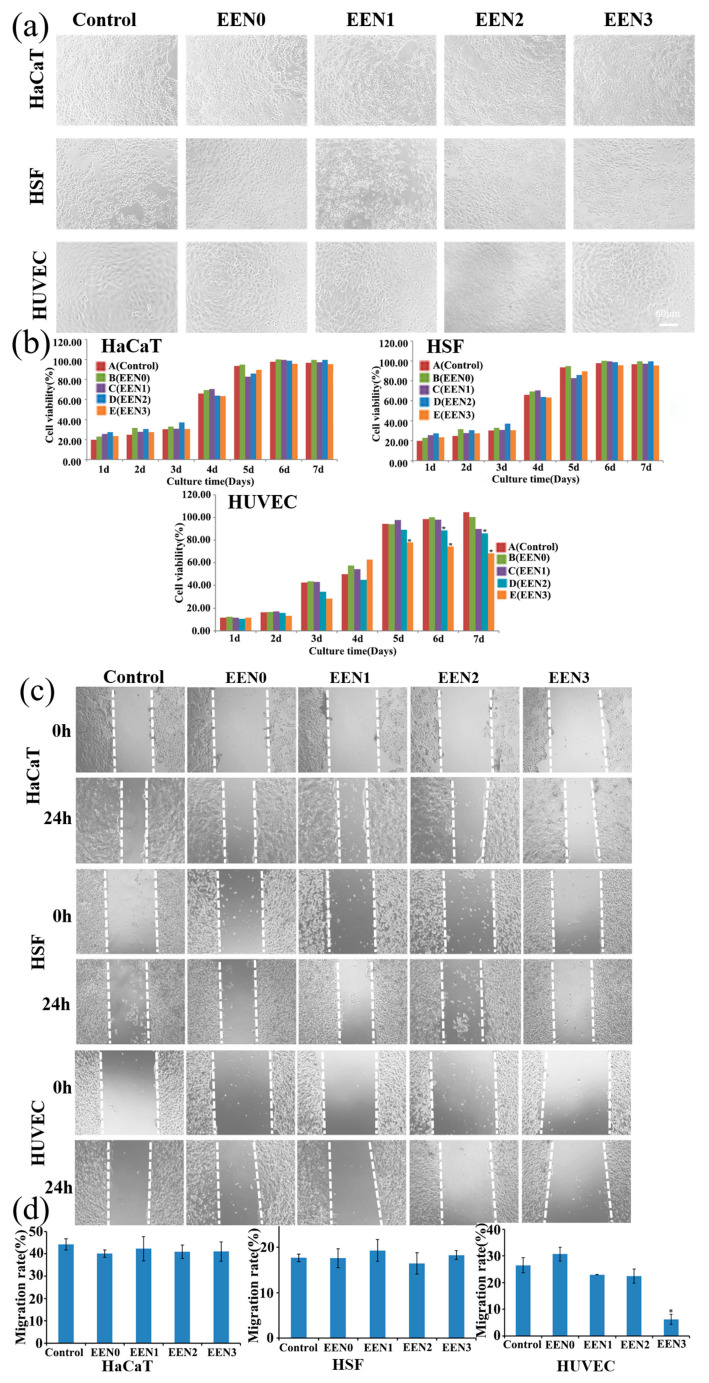
Effects of nanofibers on cell viabilities and migrations of HaCaT, HSF, and HUVEC cells. (**a**) Morphologies of HaCaT, HSF, and HUVEC cells treated with blank control, nanofibers without emodin (EEN0), nanofibers containing 0.0824 μg emodin (EEN1), 0.169 μg emodin (EEN2), 0.317 μg emodin (EEN3) for 1, 2, 3, 4, 5, 6, 7 d, respectively. Scar bars: 60 μm. (**b**) Cell viability statistics data. * *p* < 0.05 vs. control group. (**c**) Migration images of HaCaT, HSF, and HUVEC cells treated with blank control, nanofibers without emodin (EEN0), nanofibers containing 1.648 μg emodin (EEN1), 3.38 μg emodin (EEN2), 6.34 μg emodin (EEN3) for 24 h, respectively. Scar bars: 60 μm. (**d**) Migration ratio statistics data. * *p* < 0.05 vs. control group.

Ideal wound dressing should possess good biocompatibility [7]. The viabilities and migrations of HSF, HaCaT, and HUVEC cells treated with the emodin-loaded nanofibers were inspected. As shown in Figure 4a,b, HSF and HaCaT cells, after seven-day treatments with the nanofibers, exhibited similar morphologies and viabilities with the cells in the control group (*p* > 0.05), demonstrating the nanofibers had good cytocompatibility on HSF and HaCaT cells during the incubation period. Although the morphologies of HUVEC after 7 days of treatment with the nanofibers did not show clear changes, from day 5 to day 7, the viabilities of HUVEC cells treated with the nanofibers encapsulating 0.317 and 0.169 μg emodin were significantly decreased compared with the control group (*p* < 0.05), respectively, which implied that emodin inhibited the proliferation of HUVEC in a dose- and time-dependent manner, which is consistent with the reference, in which emodin inhibited VEGF-induced proliferation, migration, invasion and tube formation of HUVECs [42]. Furthermore, the results also implied that the anti-cancer activity of the nanofibers with emodin is promising based on anti-angiogenic therapy as a valuable approach to treating tumors by inhibiting endothelial cell viabilities [43].

The migration rates of HSF and HaCaT cells treated with the emodin-loaded nanofibers did not exhibit significant change (*p* > 0.05) compared with the control group, indicating that the nanofibers did not affect the migrations of HSF and HaCaT cells (shown in Figure 4c,d). Although the migration rates of HUVEC treated with the nanofibers loading 1.648 and 3.38 μg emodin were both similar to the control group (*p* > 0.05), the nanofibers with 6.34 μg emodin significantly decreased HUVEC migration rates (*p* < 0.05), such as 6.19 ± 1.89% migration rates of the nanofibers treatment compared with 26.46 ± 2.81% of the control group, demonstrating that the emodin-loaded nanofibers inhibited HUVEC migration in a dose-dependent manner, which is consistent with the proliferation assay of HUVEC and the reference [42].

### 3.4. Anti-Cancer Effects

**Figure 5:**
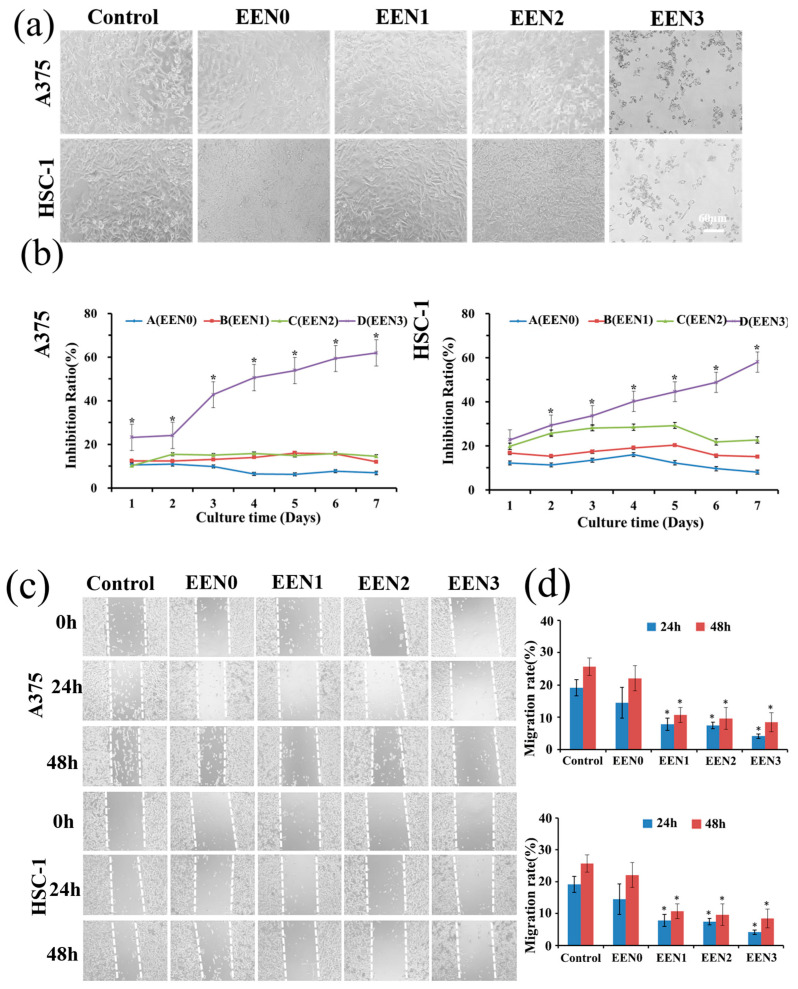

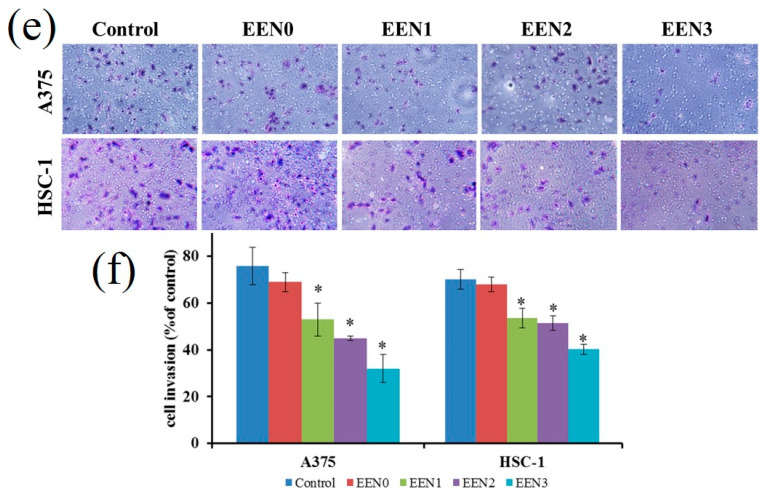
Cytotoxicity of nanofibers in A375 and HSC-1 cells. (**a**) Morphologies of A375 and HSC-1 cells treated for 7 d with the blank control, the nanofibers encapsulating 0 μg emodin (EEN0), 0.0824 μg emodin (EEN1), 0.169 μg emodin (EEN2) and 0.317 μg emodin (EEN3), respectively. (**b**) Cell viability statistics data. * *p* < 0.05 vs. the control group. (**c**) Cell migration assay of A375 and HSC-1 cells treated for 24 and 48 h with the nanofibers encapsulating 0 μg emodin (EEN0), 1.648 μg emodin (EEN1), 3.38 μg emodin (EEN2), and 6.34 μg emodin (EEN3), respectively. (**d**) Statistics data of A375 and HSC-1 cell migration rates, * *p* < 0.05 vs. the control group. (**e**) Cell invasion assay of A375 and HSC-1 cells treated for 48 h with the blank control, the nanofibers encapsulating 0 μg emodin (EEN0), 0.412 μg emodin (EEN1), 0.845 μg emodin (EEN2), and 1.585 μg emodin (EEN3), respectively. (**f**) Cell invasion statistics data of A375 and HSC-1 cells. * *p* < 0.05 vs. the control group.

After the treatments with the emodin-loaded nanofibers for 7 days, the morphologies of A-375 and HSC-1 cells gradually changed from an adherent shape to a floating shape, and the number of apoptotic-like cells remarkably increased in a dose-dependent manner (shown in Figure 5a). Meanwhile, the emodin-loaded nanofiber groups decreased the viabilities of A-375 and HSC-1 cells, especially the nanofibers with 0.317 μg emodin, significantly inhibited the viabilities of A-375 and HSC-1 cells from the first day of treatment compared with the control group (*p* < 0.05), demonstrating that the emodin-loaded nanofibers inhibited the proliferation of A-375 and HSC-1 cells in a dose-dependent manner.

The treatments with the nanofibers encapsulating 1.648, 3.38, and 6.34 μg of emodin significantly decreased the migration rates of A-375 and HSC-1 cells compared with the control group (*p* < 0.05). Treatments on A-375 cells with the nanofibers resulted in migration rates of 7.79 ± 1.88, 7.47 ± 1.01, and 4.16 ± 0.65% at 24 h, and 10.76 ± 2.35, 9.66 ± 3.42, and 8.48 ± 2.91% at 48 h, respectively, compared with 14.51 ± 4.76 and 22.07 ± 3.92% in the control group, respectively. Treatments on HSC-1 cells with the nanofibers resulted in migration rates of 12.51 ± 4.32, 7.94 ± 5.25, and 6.26 ± 2.37% at 24 h, and 14.05 ± 0.92, 8.70 ± 2.85, and 7.85 ± 1.45% at 48 h, respectively, compared with 15.10 ± 1.98 and 22.07 ± 3.92% in the control group, respectively. The results also confirmed that the emodin-loaded nanofibers exhibited anti-cancer effects by inhibiting the migrations of A-375 and HSC-1 cells.

The inhibitory effects of the emodin-loaded nanofibers on cancer cell invasion were also evaluated. As shown in Figure 5e,f, the treatments with the nanofibers encapsulating 0.412, 0.845, and 1.585 μg emodin for 48 h significantly suppressed the invasion of A-375 and HSC-1 cells compared with the control group (*p* < 0.05), indicating that the emodin-loaded nanofibers also can produce anti-cancer effects by suppressing cancer cell invasion in a dose-dependent manner.

### 3.5. Anti-Cancer Effect Mechanism

#### 3.5.1. Inhibit Cancer Cell Proliferation via Necroptosis and Apoptosis Pathway

**Figure 6:**
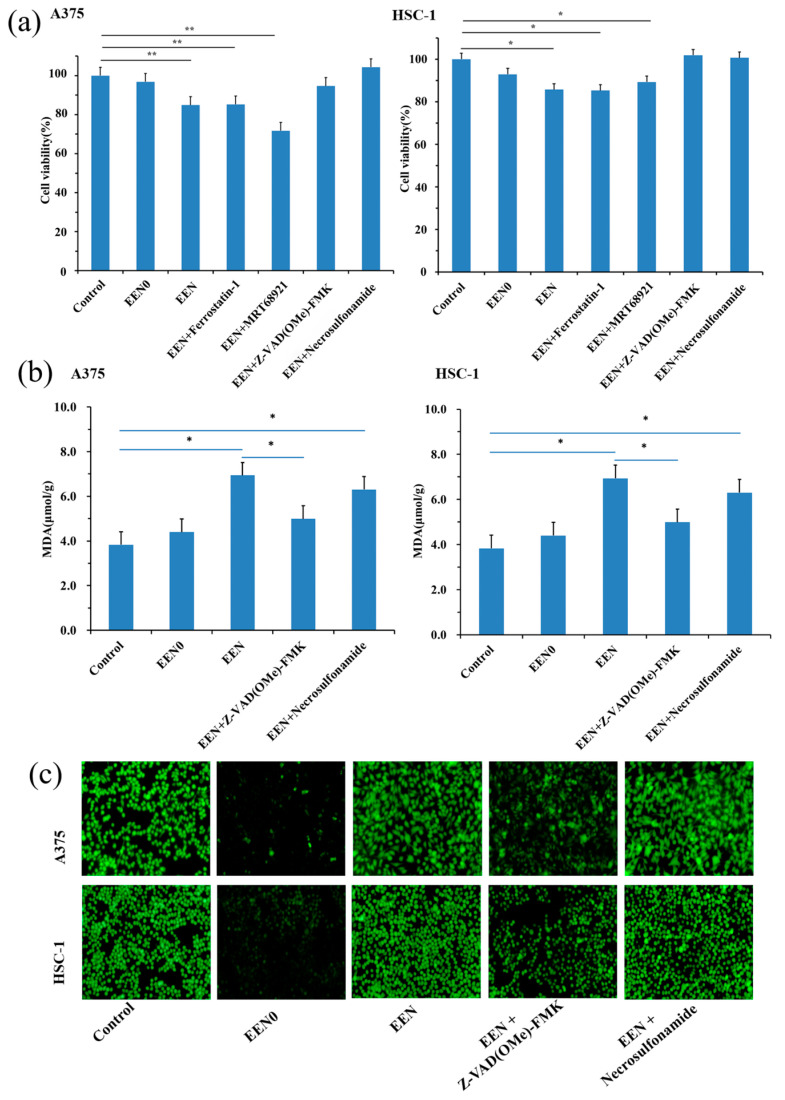
Anti-cancer mechanism of nanofibers. (**a**) Cell viabilities of A375 and HSC-1 cells, (**b**) MDA level in A375 and HSC-1 cells, (**c**) ROS level in A375 and HSC-1 cells with different treatments. * *p* < 0.05, ** *p* < 0.01 vs. the control group.

Based on the cytotoxicity of the emodin-loaded nanofiber delivery system in the cancer cells, its potential anticancer mechanism was evaluated. As shown in Figure 6, Ferrostain-1 (a ferroptosis inhibitor) and MRT68921 (an autophagy inhibitor) did not reverse the death of A-375 and HSC-1 cells induced by the emodin-loaded nanofibers, while Z-VAD(OMe)-FMK (an apoptosis inhibitor) and Necrosulfonamide (a necroptosis inhibitor) significantly reversed the death of A-375 and HSC-1 cells, demonstrating that the nanofibers with emodin could induce tumor cell death via the necroptosis and apoptosis pathway, which is consistent with the reference [42].

#### 3.5.2. MDA Level in Tumor Cells

MDA is one of the final products of polyunsaturated fatty acids peroxidation in the cells, and MDA level is commonly known as a marker of oxidative stress [44]. The nanofiber treatment significantly increased the MDA level compared with the control group (*p* < 0.05), while Z-VAD(OMe)-FMK significantly reversed the enhanced MDA level induced by emodin, which demonstrated that the nanofibers induced the death of A-375 and HSC-1 cells related to promoted MDA production by apoptosis pathway. The reversed effects of Necrosulfonamide on MDA level were not significant compared with the alone nanofiber treatment group (*p* > 0.05), suggesting that the emodin-loaded nanofiber-induced death of A-375 and HSC-1 cells did not correlate with MDA production via the necroptosis pathway.

#### 3.5.3. ROS Level in Tumor Cells

Oxidative stress (OS), characterized by excessive accumulation of ROS, is an emerging hallmark of cancer; the aberrant increase in intracellular ROS levels can cause cell death because of nonspecific oxidation damage to key cellular biomolecules [45,46]. The nanofiber treatment significantly increased ROS levels compared with the control group (*p* < 0.05, Shown in Figure 6c), suggesting that emodin inducing the death of A-375 and HSC-1 cells might relate to the ROS pathway. Furthermore, Z-VAD(OMe)-FMK significantly reversed the enhanced ROS level induced by emodin, implying that the nanofibers induced the death of A-375 and HSC-1 cells related to promoted ROS production by apoptosis pathway, which is consistent with the reference [42]. While the reversed effects of Necrosulfonamide on ROS levels were not significant compared with the alone nanofiber treatment group (*p* > 0.05, Shown in Figure 6c), suggesting that the emodin-loaded nanofiber-induced death of A-375 and HSC-1 cells did not relate to ROS overproduction via the necroptosis pathway.

### 3.6. Anti-Bacterial Activities

**Figure 7:**
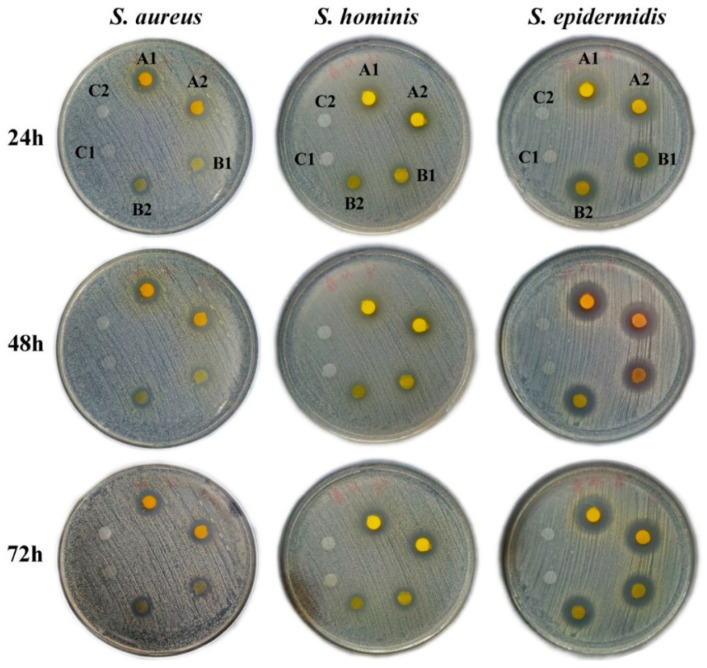
Anti-bacterial effects of nanofibers. A1–A2, B1–B2, and C1–C2 denoted the nanofibers encapsulating 210, 30, and 0 μg of emodin, respectively.

The results of the anti-bacterial experiment showed that the nanofibers encapsulating 30 and 210 μg of emodin exhibited significantly inhibitory effects on *S. aureus*, *S. hominis,* and *S. epidermidis* in a dose-dependent manner (shown in Figure 7). The anti-bacterial zones against *S. aureus*, *S. hominis,* and *S. epidermidis* were 12 ± 0.43, 12 ± 0.43, and 15 ± 0.50 mm after 24 h treatment with the nanofibers with 210 μg emodin, respectively, following, reached to 13 ± 0.50, 13 ± 1.12, and 17 ± 0.43 mm after 72 h treatment, respectively. While the antibacterial zones treated with the nanofibers with 30 μg emodin were smaller, illustrating that the anti-bacterial effects were in a dose-dependent manner. Moreover, with an increase in treatment time, the anti-bacterial zones against the three types of bacteria gradually increased, which further demonstrates the long-lasting anti-bacterial effects of the emodin-loaded nanofibers, which is consistent with the biphasic release profile of emodin from the nanofibers. 

## 4. Conclusions

We developed emodin-loaded electrospinning nanofibers with anti-cancer and anti-bacterial multifunctional bioactivities, which can provide a potential strategy for postoperative management of skin cancer. The nanofibers encapsulating emodin with a smooth surface exhibit a uniform diameter distribution and a clear shell-core structure. Meanwhile, emodin as a crystal and hydrophobic compound was evenly dispersed in the nanofibers, which significantly enhanced the solubility of emodin in an acidic medium, which is beneficial for its anti-cancer effects in acidic tumor microenvironments [25]. The emodin-loaded nanofibers not only display good wettability, high entrapment efficiency, and biphasic release profile but also present expectant biocompatibility. They especially displayed anti-cancer properties by increasing the levels of MDA and ROS in cancer cells via the apoptosis pathway, as well as long-term anti-bacterial effects in a dose-independent manner. To sum up, the emodin-loaded nanofiber delivery system can provide a potential strategy for postoperative management of skin cancer.

## Data Availability

The data presented in this study are available on request from the corresponding author.
